# Supplementary material for: We know what we need: Older adults’ and stakeholders’ perspectives on ageing, health, and wellbeing in Pakistan
Source: PLOS Glob Public Health. 2026 Feb 20;6(2):e0005916. doi: 10.1371/journal.pgph.0005916 (PMC12923039; doi:10.1371/journal.pgph.0005916)
Supplement: S1 File — (PDF) [file pgph.0005916.s001.pdf]

We Know What We Need: Older Adults' and Stakeholders' Perspectives on Ageing, Health, and Wellbeing in Pakistan

Supporting Information – S1 File

S1: Participant focus group discussion (FGD) guide.

Questions	Probes
Bio Data	Type of interviewee: Older Adult / Caregiver
	Location: Urban / Rural
According to you, what is aging? How would you define it?	How do you feel about growing older?
What are the challenges or hardships you face in aging?	Are these challenges related to health, mobility, finances, or relationships? Which ones affect you most?
What are your priorities as you get older?	For yourself (e.g. health, comfort), for your family, and for the government or society
	What do you wish for?
What are the main obstacles and enablers for achieving or maintaining your priorities?	What support do you already have?
	What additional support do you need from family, friends, or community?
Individual Level: What helps you stay active and independent?	What barriers make it difficult?
Interpersonal Level: How is your relationship with your family or caregiver?	Do you feel supported by them?
Community Level: Are there places or groups in your community that support older adults?	For example, community centers, mosques/churches, local organizations.
	Do you participate in any?
Societal Level: What do you think society or the government should do to improve the lives of older people?	What kinds of programs or facilities do you think are needed?
What changes would make your daily life easier?	For example, transportation, safety, social inclusion, or healthcare access.
Is there anything else you want to share about aging or what could make it better for you?	Any final message or suggestion for families or policymakers?
What areas would you like to see researched?	Is there any issue which needs to be explored?
