# Supplementary material for: We know what we need: Older adults’ and stakeholders’ perspectives on ageing, health, and wellbeing in Pakistan
Source: PLOS Glob Public Health. 2026 Feb 20;6(2):e0005916. doi: 10.1371/journal.pgph.0005916 (PMC12923039; doi:10.1371/journal.pgph.0005916)
Supplement: S3 File — (PDF) [file pgph.0005916.s003.pdf]

Theme (SEM Level)	Theme type	Participant type	Finding
1st	Problem	Older	Weakness
1st	Solution	Older	Need for financial assistance
1st	Problem	Older	Stress
1st	Problem	Older	Lack of resources depsite skills to earn
1st	Problem	Older	Salaries not being offered that proper time
1st	Solution	Older	Need for affection from family
1st	Problem	Older	Delay in payment of wages
1st	Problem	Older	Self-dependency
2nd	Problem	Older	Lack of family support
3rd	Problem	Older	Children's nature (Obedient/Spolied)
1st	Problem	Older	Unable to visit people
1st	Problem	Older	Basic Nessecities
1st	Solution	Older	Need for wheelchair
1st	Solution	Older	Need for better roadside system
1st	Problem	Older	Presence of fake medicine

1st	Solution	Stakeholder	Need of supportive children
1st	Solution	Stakeholder	Need for time from children
1st	Problem	Stakeholder	Advisory committee
1st	Solution	Stakeholder	Need of old homes
1st	Solution	Stakeholder	Need of resources to keep oneself engaged
1st	Solution	Stakeholder	Need for support from neighbourhood/NGO/Gove rnment
1st	Problem	Stakeholder	Lack of nearby health care facility
1st	Problem	Stakeholder	Poor quality first aid facility
1st	Problem	Stakeholder	Poor roads
1st	Problem	Stakeholder	Water supply

2nd	Problem	Older	Mindset
2nd	Problem	Older	Overthinking
2nd	Problem	Older	Loneliness / Fear of Death
2nd	Problem	Older	Timing of meals
2nd	Problem	Older	Urbanization
2nd	Problem	Older	Presence of pure/organic food produce
2nd	Problem	Older	Morality
2nd	Problem	Older	Electricity and water maintainance
2nd	Problem	Older	Education of children

Quotes
As people age, they experience weakness and become more susceptible to disease. Their weakness worsens gradually; they lose courage and willpower with age.
We want your organization to provide us with financial aid… we do not have enough strength to make money on our own. We need external support.
Poverty leads to interpersonal stress. Children grow tired of us after marriage.
We don't have enough resources to make money with the skills we already have. Many of us are good at sewing and embroidery, but do not have our own sewing machines.
We even do labor work, but people do not pay us, then what do we do?
Individuals are helpless, but where do we eat from ?
If there is love and affection from the children, then life can be lived happily even without money.
When we have a source of income, then we are not worried, but when we do not have a source of income, then we are worried. Where do I get the money form, nobody even lends it, nobody is even willing to give five rupees to someone. We don't get our pay; it has been five months.
We are poor, we make our flatbread, we break the flatbread into two pieces, give half to the beggar and keep half for ourselves
If an old person gets paralyzed due to illness, their relatives, their children, they themselves turn sullen. After that, if that old person, in their state of illness, are unable to receive proper treatment and medication, then they may experience more stress and difficulty.
If, however, the person's children are spoiled and uncaring, then that contributes to further stress and worry on their mind. If the child is obedient then the old person can, to some extent, find some encouragement and support in their illness, disputes, etc.
Whenever there is a wedding, funeral, or I wish to go pay my respects and condolences to someone, I cannot even go there. Relatives, dear ones, strangers, I cannot go to meet anyone.
in terms of facilities, they are the same as those that I mentioned earlier like the availability of electricity, water, and gas. Other than this, having a stable and good income is important.
Those old people who are very weak and malnourished must have wheelchairs available to them.
The road system outside houses should be good and the washroom, etcetera, inside the house for old people should be good and have the facility of a commode available
The medicines that are available nowadays can be fake too.

Children should serve their old parents. They should take care of their parents' diet especially. If they are ill/unwell (have an illness, etcetera) then children should make arrangements for their treatment. They should act on their responsibility. All efforts should be made to ensure that they remain healthy and well

children should give as much time to their parents as possible. Giving them time makes them lead a quite peaceful and happy life.

Other than that, the government should establish institutions for people aged 60 years and above where only people who have retired will pool in their ideas on the basis of their experiences so that they can also become valued citizens.

M: There should an old house. How many people agree with my statement?

Four people agree.

M: Cleanliness? How many people agree with this statement of mine?

Four people agree.

M: Medical coverage should be free.

Five people agree

M: A counseling meeting should be arranged. How many people agree with this statement?

Two people agree.

M: There should be a good and healthy diet. What percentage of people agree with my statement?

5 people agree.

M: Medication/treatment should be free. How many people agree with this?

4 people agree.

M: There should be wheelchairs for disabled individuals. How many people agree with this statement?

8 people agree.

According to us there should be an AC, TV, etcetera. And a newspaper should also be available on a daily basis.

Neighbors should take care of their old neighbors. On a community level, an NGO should be established that will serve to help old people financially. On a government level, public ambulances are available as are pensions. Access to electricity, water, and gas should be there.

C: The absence of a medical clinic in our village is a source of stress for us. There is a medical store also.

In our village, the facility of first aid for old people is of poor quality.

There is a transport issue. Cars reaching late due to the poor condition of roads.

we do not have a good water supply. The water from the water supply is not right (of good quality). Other than that, medication is quite expensive in hospitals.

A happy person gets old late as compared to a sad person who gets old earlier

when a person retires, he stays at home. In anxiety and poverty, man becomes overthinker.
The problems and tensions have increased, relatives have left us. Our blood (relatives) left us, youthfulness left us, slowly everyone just left us like that. Fear of death is also there. Someone's mother dies someone's father and we don't only have children and family ahead us, there is a 'hereafter' ahead us. Many hardworking people have departed.
Getting food at the right time and along with this, doing exercises. Food should be appropriate according to time, in appropriate amounts, also do exercise, keep himself happy. If they just sit after eating, they will become weak. Leisure activities are also necessary, they need food, they need happiness, they want to stay happy
nowadays people have low life expectancy, earlier people used to have longer lives. When food used to be organic. Now, there is just artificial food which results in decreased life. People used to live for 100-150 years. They used to do exercise, used to walk by foot. Their diet was less but healthy.
food should be pure. In the past there were crops which had the power of soil. We used to eat pure. There used to be vitamins in our food.
4) there are poisonous medicines now.
when a person is good, he has spiritual peace. It is correct that this increases life. If he is not a good man, he will have problems and his life-span will be reduced. According to Islam if a man follow the righteous path, he will have spiritual peace and remain happy even if he is hungry.
here transformer was burned and wasn't replaced for days (because of rain), there was no water for 3 months
free education for children is also needed. Our children are also part of our lives, we need free education for them as well
