# Supplementary material for: We know what we need: Older adults’ and stakeholders’ perspectives on ageing, health, and wellbeing in Pakistan
Source: PLOS Glob Public Health. 2026 Feb 20;6(2):e0005916. doi: 10.1371/journal.pgph.0005916 (PMC12923039; doi:10.1371/journal.pgph.0005916)
Supplement: Attachment — Submitted filename: Response_SS_22102025.docx [file pgph.0005916.s005.docx]

Dear Editor,

Thank you for sharing the Reviewer’s comments. Here is our response:

| **Comment** | **Response** |
| --- | --- |
| The introduction is well written and refers to the topic at hand. However, I would suggest a redesign of some phrases to improve the flow of the "story". The transition from the first paragraph to the second (line 83-84) comes very sudden and needs a sentence that transfers the focus from the challenges mentioned to the social determinants of health. The paragraph starting from line 95 deals with the reasoning for this article and I would put this rather towards the end as it is a perfect predecessor for the aim of the article. Overall, the introduction needs a bit more structure, alignment and logical flow rather than jumping from one topic to another. | Thank you for this comment. We have added transition sentences across the introduction to make the transitions between the paragraphs appear less sudden. Additionally, we have also restructured the introduction a bit to make the text flow better. |
| - In the methods section, you briefly mentioned the broader multi-method research project, please also indicate the name (and any Grant Agreement Number or similar ID), so that interested readers can look it up. You also mentioned in the data collection to refer to the main study. Readers cannot do that without knowing the main study. | This qualitative study was nested within the broader multi-method project titled *Exploring the priorities of ageing populations in Pakistan, comparing views of older people in Karachi City and Thatta* published in PLOS ONE (DOI: [10.1371/journal.pone.0304474](https://doi.org/10.1371/journal.pone.0304474)). The main study aimed to identify the needs and priorities of older adults and stakeholders across urban and rural locations, exploring their views on the definition of ageing, areas of importance, available services, and barriers to living well in later life. The current paper extends this work by thematically examining barriers and solutions to healthy ageing using data from FGDs conducted under the same study framework. (Line 113-120)  As for funding, this study was supported by the Institute of Global Innovation, University of Birmingham, UK. The award was received by Justine Davies and Carolyn Greig, with no formal grant number as the funding was internal. Further details about the Institute and its global research themes are available at <https://www.birmingham.ac.uk/research/global-goals/igi/index.aspx>. The funders had no role in study design, data collection and analysis, decision to publish, or preparation of the manuscript. (Line 545-549)  We have incorporated all the details stated above in the manuscript. |
| - Regarding the recruitment of older adults, it would be beneficial to state a few details on the recruitment process. How did the community mobilizer and research coordinator contact these people (lines 143-147)? | Thank you for this helpful suggestion. We have now elaborated on the recruitment process in the *Methods – Study Setting, Population, and Recruitment* section. The revised text explains that the community mobilizer, a trusted local representative, contacted potential participants through household visits, word-of-mouth communication, and engagement with local leaders and neighborhood committees. The research coordinator subsequently followed up with interested individuals, confirmed eligibility, and scheduled participants for workshops. The updated paragraph also clarifies that referrals among participants were used to identify additional eligible individuals. (Line 132-139) |
| - Please also state any software or programs used (lines 178-179) | All analyses and data management were conducted using Microsoft Excel (Microsoft Corporation, Redmond, WA, USA). (Line 185-186) |
| - You referred to the studies four main research questions (line 200) without mentioning them before. Please add them where appropriate (e.g. together with the study aim at the end of the introduction) | Thank you for this important suggestion. We have added the main research questions at the end of the introduction section. (Line 119-126) |
| - I would also suggest to include a legend for table 1 (below). Readers may be able to proceed the bulk of information better, if barriers and solutions are placed in columns next to each other instead of rows. It makes it easier to compare and contrast (Column 1: Level of framework, Column 2: Themes Barriers, Column 3: Themes solutions) | Thank you for this suggestion. The columns have been re-arranged in table 1 as per the suggestion. (Line 200) |
| - The figure is very appealing, but I am wondering what the message or added value behind it should be? It only focuses on the concerns and I think the value of the paper would be increased a lot to also highlight the solutions. Please think about redesigning the figure also integrating (and emphasizing) the solutions! | Thank you for this suggestion. The figure has been redesigned to sequentially discuss the barriers, proposed mechanisms of change as well as solutions, for both urban and rural settings. (Line 400) |
| - The discussion offers an appropriate reflection of the results with contemporary literature. There are two citations missing: Mazumder et al. (lines 461-466) and Michigan Study (lines 494-500). | Thank you for highlighting this discrepancy. We have added both citations. |
| - What I am completely missing - which in my opinion is an absolute essential part of every scientific rigorous publication - is the critical reflection of the strengths and limitations of your work. Please include this in the discussion. E.g. a limitation coming to my mind is that only one reviewer conducted the analysis/coding (as mentioned in line 195), which may affect researcher bias and interpretive rigor, amongst others. Please critically go through your work and elaborate on the strengths and limitations of your methodology as well as a reflexivity part in the discussion! | We have added a dedicated section discussing the study’s Strengths and Limitations and expanded the Researchers’ Reflexivity paragraph in the Discussion to provide a more critical and transparent reflection of our methodology.  The reflexivity section has also been expanded to acknowledge the research team’s positionality, the involvement of local moderators fluent in Sindhi and Urdu, and steps taken to preserve authenticity and participant voice. |
| - It would also be great to provide the semi-structured guide for the focus groups as supplementary material. Even the coding process would be very interesting for readers. | Thank you for this important comment. We have provided the FGD guide in the supplementary information document and included details about the coding process in the manuscript. (Line 177-186) |
| All PLOS journals now require all data underlying the findings described in their manuscript to be freely available to other researchers, either  1. In a public repository,  2. Within the manuscript itself, or  3. Uploaded as supplementary information. | All data underlying the findings described in this manuscript are fully available upon request so as to not breach the institutional ethical review board. The FGD guide material has been provided as supplementary Information files. |
